# Synthesis and Biological Evaluation of a Radiolabeled PET (Positron Emission Tomography) Probe for Visualization of In Vivo α-Fucosidase Expression

**DOI:** 10.3390/ph14080745

**Published:** 2021-07-29

**Authors:** Jonathan Cotton, Chris Marc Goehring, Anna Kuehn, Andreas Maurer, Kerstin Fuchs, Bernd J. Pichler

**Affiliations:** 1 Werner Siemens Imaging Center, Department of Preclinical Imaging and Radiopharmacy, Eberhard Karls University of Tübingen, 72076 Tübingen, Germany; chris-marc.goehring@student.uni-tuebingen.de (C.M.G.); anna-kuehn82@gmx.de (A.K.); andreas.maurer@med.uni-tuebingen.de (A.M.); fuchs.kerstin@googlemail.com (K.F.); bernd.pichler@med.uni-tuebingen.de (B.J.P.); 2 Cluster of Excellence iFIT (EXC 2180) “Image Guided and Functionally Instructed Tumor Therapies”, Eberhard Karls University of Tübingen, 72076 Tübingen, Germany

**Keywords:** α-fucosidase, PET tracer, ^11^C, cancer, inflammation

## Abstract

The acidic hydrolase α-fucosidase (AF) is a biomarker for maladies such as cancer and inflammation. The most advanced probes for α-fucosidase are unfortunately constrained to ex vivo or in vitro applications. The in vivo detection and quantification of AF using positron emission tomography would allow for better discovery and diagnosis of disease as well as provide better understanding of disease progression. We synthesized, characterized, and evaluated a radiolabeled small molecule inhibitor of AF based on a known molecule. The radiosynthesis involved the ^11^C methylation of a phenoxide, which was generated in situ by ultrasonification of the precursor with sodium hydride. The tracer was produced with a decay corrected yield of 41.7 ± 16.5% and had a molar activity of 65.4 ± 30.3 GBq/μmol. The tracer was shown to be stable in mouse serum at 60 min. To test the new tracer, HCT116 colorectal carcinoma cells were engineered to overexpress human AF. In vitro evaluation revealed 3.5-fold higher uptake in HCT116AF cells compared to HCT116 controls (26.4 ± 7.8 vs. 7.5 ± 1.0 kBq/10^6^ cells). Static PET scans 50 min post injection revealed 2.5-fold higher tracer uptake in the HCT116AF tumors (3.0 ± 0.8%ID/cc (*n* = 6)) compared with the controls (1.2 ± 0.8 (*n* = 5)). Dynamic scans showed higher uptake in the HCT116AF tumors at all time-points (*n* = 2). Ex vivo analysis of the tumors, utilizing fluorescent DDK2 antibodies, confirmed the expression of human AF in the HCT116AF xenografts. We have developed a novel PET tracer to image AF in vivo and will now apply this to relevant disease models.

## 1. Introduction

Tools that can non-invasively detect metabolic alterations in vivo provide us with unique and profound opportunities in personalized medicine and offer insight into the cause, progression, and treatment of disease. Positron emission tomography (PET) is an imaging modality that offers high sensitivity and good spatial resolution, relying on the administration of radiolabeled biologically active molecules that can be detected in trace quantities. Through the use of PET probes, biological processes can be interrogated without effecting changes in the system being evaluated.

The acidic hydrolase α-fucosidase (AF) has been connected to many diseases. AF is responsible for the digestion of terminal α-linked fucose residues from polysaccharides and glycoconjugates, showing high selectivity toward its substrate. Increased AF expression can be associated with inflammation [1], cystic fibrosis [2], cancer [3,4,5,6], and *Helicobacter pylori* infection [7]. AF mRNA expression levels in certain breast tumors have been shown to be more than 139-fold higher than that observed in normal breast tissue samples. The high AF mRNA levels in early-stage breast cancer correlate to a decreased cell-surface fucosylation. Upon progression to late-stage breast cancer, AF mRNA levels decrease, giving rise to higher surface fucosylation and enhanced malignancy and invasiveness. The quantification of AF provides valuable prognostic information about the disease progression and patient survival [3]. The progression of colorectal carcinoma is associated with decreases in AF expression. AF mRNA expression levels in advanced CRC tumors can be up to 60% lower than the levels expressed in healthy mucosal tissue [4]. It has also been shown that AF is a useful biomarker for detecting hepatocellular carcinoma (HCC). The serum AF activity level in patients with HCC was found to be more than 2-fold higher than that in patients with cirrhosis or other neoplasms, or in the healthy population [5,6]. Despite its intricate and substantial involvement in disease, there have been no published reports of AF-specific in vivo imaging agents. We postulated that a radiolabeled inhibitor of AF would be suitable for such imaging and provide substantial benefit to the imaging community.

**Figure 1:**
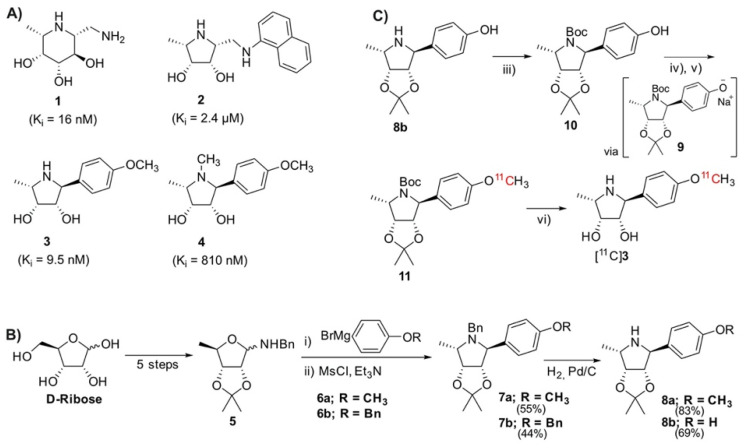
(**A**) Structures and K_i_ values of known inhibitors of α-fucosidase [8,9,10]; (**B**) Overview of the synthetic route to core pyrrolidine structure from D-ribose [10]; (**C**) Synthetic route toward precursor **10** and subsequent radiosynthesis of [^11^C]**3** via the phenoxide **9** and radiolabeled intermediate [^11^C]**11**. Reagents and conditions: (iii) Boc_2_O, Et_3_N, DCM, RT. (iv) NaH, CH_3_CN, ultrasonification, 5 min, RT. (v) [^11^C]CH_3_I, CH_3_CN, 110 °C, 5 min. (vi) 6M HCl, 140 °C, 5 min.

There are many reported antagonists of AF (Figure 1A) with varying inhibitory ability. Fuconojirimycin **1** is a well-known inhibitor but is synthetically challenging. Molecules built around a pyrrolidine core, e.g., **2**, **3,** and **4**, have recently shown potential as selective AF inhibitors [8,9,10]. At physiological pH, the protonated azasugar, which can mimic the oxocarbenium intermediate of the natural substrates, binds strongly to the enzyme’s active site [11]. The importance of the secondary amine for inhibition is demonstrated through the *N*-methylation of **3** to **4**, resulting in an 81-fold increase in K_i_. The unnatural pyrrolidine **3** was thus chosen as it possesses favorable, well established pharmacological properties, i.e., an IC_50_ value of 58 nM and more importantly, a K_i_ of 9.5 nM. Notably, molecule **3** contains a methoxyphenyl moiety, which was thought to be a good candidate for late-stage methylation with [^11^C]CH_3_I to produce an isotopically labeled probe with proven pharmacology. 

Given the wide range of pathologies associated with changes in AF expression, a radiolabeled small molecule inhibitor of AF was envisaged, which could be used to probe AF expression in preclinical and clinical disease models. Compared to a substrate, which is useful for quantifying enzyme activity, the radiolabeled inhibitor binds tightly to its associated enzyme, allowing one to not only show localization of AF, but also provide quantitative information about enzyme expression at the protein level. The aim of this study was to establish a robust and rapid synthesis of [^11^C]**3** and evaluate its potential for detecting AF expression in vivo using PET.

## 2. Results and Discussion

### 2.1. Chemical Synthesis of Precursor and Optimization of Deprotection

Access to the reference title compound **3** and the family of pyrrolidines outlined by Kotland relies on synthesis of the glycosylamine **5**, which can be produced in five steps from commercially available D-ribose (Figure 1B) [10]. A reaction of **5** with carbon nucleophile **6a** yields an amino-alcohol, which undergoes an intramolecular cyclization after mesylation to give **7a** [10]. The *N*-benzyl moiety is removed by palladium-catalyzed hydrogenation, producing **8a**.

We envisioned a late-stage ^11^CH_3_I methylation to facilitate an isotopic exchange of the methoxy-methyl of **3**, wherein access to the free phenol moiety was required. Various attempts to cleave the phenolic ether of **7a** were made using BBr_3_ [12], AlI_3_ [13]_,_ and AlCl_3_ [14]. Unfortunately, the desired chemoselectivity could not be achieved, as evidenced by the simultaneous cleavage of the acetonide with the phenolic ether, along with many unidentified side-products. We contemplated introducing a masked phenol moiety by reacting the benzylamine **5** with the Grignard **6b**, containing an *O*-Bn protected phenol. Kotland’s methodology proved robust, with the reaction successfully producing **7b**. In agreement with the literature data, the ^1^H NMR analysis revealed the expected 14 aromatic protons (Figure S1, Supplementary Materials). The structure was further elucidated through a doublet of 2.4 Hz at 3.98 ppm, corresponding to H-1 [10]. The *N*-benzylic protons appeared as two distinct doublets at 3.65 and 3.28 ppm, with strong geminal coupling of 14.2 Hz, reflecting their proximity to the chiral environment of the pyrrolidine. The *O*-benzylic protons appeared as a singlet at 5.06 ppm, reflecting their magnetic isolation from the rest of the molecule. Both benzyl moieties (*O*-Bn and *N*-Bn) were simultaneously debenzylated to give the intermediate **8b**, which was evinced by the disappearance of 10 aromatic and 4 benzylic resonances in the ^1^H NMR spectrum (Figure S2, Supplementary Materials).

To prevent nucleophilic competition, the secondary pyrrolidine amine was protected to allow for the final ^11^CH_3_I *O*-methylation (Figure 1C). This was achieved through the *N*-Boc protection, to the precursor **10**. Even after multiple attempts to purify and dry, the complex ^1^H and ^13^C NMR spectra persisted (Figure S3, Supplementary Materials). This may be due to the molecule existing as a rotamer, presumably resulting from steric crowding between the phenol and carbamate, preventing free rotation about the N-C bond. High resolution mass spectrometry was used to further evidence the formation of the desired product (Figure S4, Supplementary Materials).

**Figure 2:**
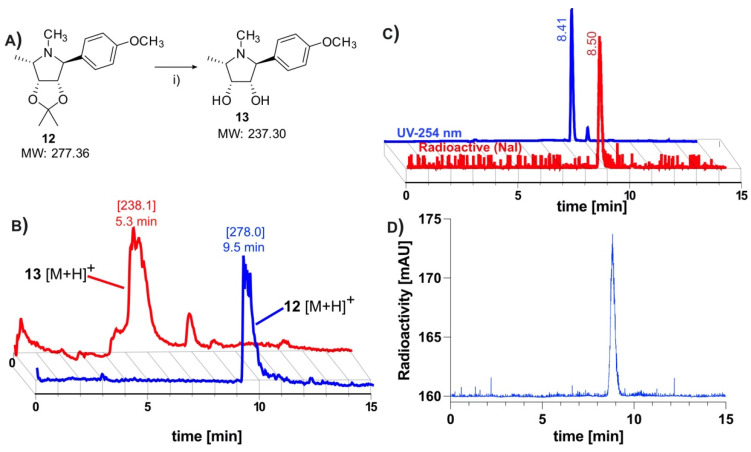
Hydrolysis of acetonide, confirmation of radiochemical identity and serum stability of [^11^C]**3**: (**A**) Synthetic scheme depicting rapid hydrolysis of acetonide-protecting group. Reagents and conditions: (i) 6M HCl, 140 °C, 5 min; (**B**) LCMS data depicting successful cleavage of acetonide-protecting group. The mass peak of the model compound **12**, which is observed at 9.5 min, disappears after heating in 6 M HCl. A new mass peak, representing the more polar deprotected product **13** is observed at 5.3 min, indicating successful removal of acetonide protection [10]; (**C**) Retention time of tracer (radio-trace) at 8.4 min correlates to non-radioactive standard (UV 254 nm) at 8.5 min; (**D**) HPLC analysis * of tracer incubated in serum at 37 °C for 1 h shows no apparent metabolites. (* Data subjected to median filter).

The removal of the acetonide protection has been described as problematic, requiring a slow hydrolysis in 1 M HCl [10]. Owing to time constraints of the short-lived ^11^C-isotope, it was first necessary to find conditions for a rapid deprotection (Figure 2A). The deprotection was evaluated using the model compound **12**, which had been prepared using Kotlands’ method [10]. Applying the conditions used by Steiger et al. [15], this was easily achieved by adding 0.15 equivalents of 6M HCl to the solution of **12** in acetonitrile (Figure 2A), followed by aggressive heating for 5 min. LCMS (Figure 2B) revealed an almost quantitative conversion of **12** ([M + H]^+^_(theor.)_ = 278.37) to **13** ([M + H]^+^_(theor.)_ = 238.31). 

### 2.2. Radiolabeling and Automation of Tracer Synthesis 

The ^11^CH_3_I methylation was nontrivial, with many conventional bases being found to be unsuitable for the radiolabeling. Cs_2_CO_3_, K_2_CO_3_, and 1,8-Diazabicyclo(5.4.0)undec-7-ene (DBU) all yielded none of the desired radiolabeled intermediate **11**, while NaOH produced only trace amounts. Although phenols are readily methylated, this was not found to be the case with our precursor. Instead of directly labeling the phenol, our approach relied on methylating an in situ generated phenoxide **9**, which was produced through room temperature ultrasonication of **10** with NaH in acetonitrile for 5 min (Figure 1C). The suspension was transferred directly into the reactor of the FX-M module. An additional advantage offered by this approach was that this step is not under the time constraints of a normal ^11^C-synthesis. Using this technique, the radiolabeling proceeded smoothly, producing the desired molecule in good yields. The crude material was diluted with an acetate buffer and subsequently subjected to semi-preparative HPLC purification (S5). To avoid the associated time penalties and hassle of further concentration steps, we aimed to find a biocompatible HPLC eluent so that the tracer could be used directly for imaging. Luckily, this was made possible by using a 10% solution of ethanol in PBS (buffered to pH 6.0). This afforded clean separation of the tracer from the precursor, which had respective retention times of 3.3 and 6.3 min. The tracer was produced with an overall decay-corrected yield of 41.7 ± 16.5% (*n* = 5), calculated from the MeI delivered to the reactor of the FX M module. The molar activity was determined to be 65.4 ± 30.3 GBq/μmol (*n* = 14). The identity of the tracer was confirmed by comparing its HPLC retention with that of non-radioactive **3** (Figure 2C). HPLC analysis of the tracer incubated for 60 min in serum revealed no apparent radio-metabolites (Figure 2D).

### 2.3. In Vitro Evaluation of Tracer

#### 2.3.1. Producing AF Overexpressing Cell Line 

**Figure 3:**
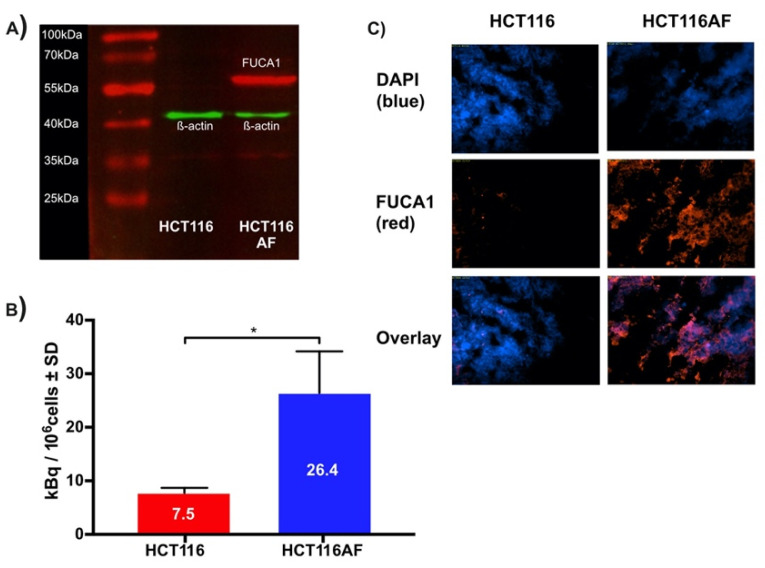
Validation of in vitro tracer uptake and expression of AF in HCT116AF and xenograft tissue: (**A**) Western blot showing expression of AF in transfected HCT116 cells. Protein bands were stained with primary anti-DDK2 and Alexa Fluor 555 labeled fluorescent secondary antibodies; (**B**) In vitro evaluation of tracer uptake revealed 3.5-fold higher tracer accumulation in HCT116AF vs. HCT116 cells, with respective uptake values of 26.4 ± 7.8 and 7.5 ± 1.0 kBq/10^6^ cells (*p* < 0.05); (**C**) Ex vivo analysis of HCT116AF tumors revealed positive staining for DDK2, indicating that the transfected cells express AF in vivo (* *p* < 0.5).

HCT116 colorectal carcinoma cells were transfected via lipofection with cDNA for human AF to produce the HCT116AF cell line. The starting cell line was chosen as it shows low endogenous expression of AF and would offer higher contrast as a control [4]. The monoclonal colonies of positively transfected cells were isolated, wherein western blot (Figure 3A) of the transfected cell lysate revealed a protein band of around 55 kDa, confirming that exogenous AF was being expressed.

#### 2.3.2. In Vitro Evaluation of Tracer 

For in vitro evaluation, HCT116AF and HCT116 cells were incubated with the tracer and then subsequently examined in biodistribution tubes using a γ-counter (Figure 3B). The uptake in HCT116AF was shown to be 3.5-fold higher than that of the untransfected HCT116 cells, with respective values of 26.4 ± 7.8 and 7.5 ± 1.0 kBq/10^6^ cells.

#### 2.3.3. Ex Vivo Evaluation of Tumors

Tumor sections were examined ex vivo to assess whether the AF protein could be detected through its DDK tag. The strong fluorescent signal in the transfected tumors and its absence in the controls confirmed that the xenografts were expressing exogenous AF in vivo. The tumor sections were thus incubated with primary antibodies against the DDK2 motif contained in the cDNA construct and stained with an appropriate fluorescent secondary antibody (Figure 3C, red channel). 4′,6-diamidino-2-phenylindole (DAPI) was used as a counterstain to visualize the cell nuclei (Figure 3C, blue channel). HCT116AF tumor sections were easily distinguishable from the HCT116 sections by their strong DDK2 signal.

#### 2.3.4. In Vivo Evaluation of Tracer

**Figure 4:**
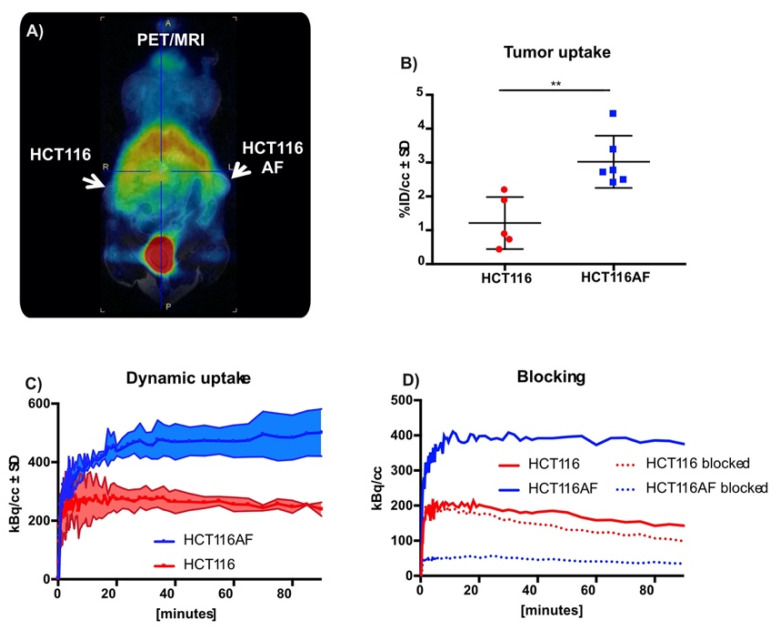
In vivo static and dynamic PET imaging of AF expression: (**A**) PET images of mouse with HCT116 (left) and HCT116AF (right) tumors show higher tracer accumulation in the AF overexpressing tumor compared to the HCT116 controls; (**B**) Quantification of tracer uptake in tumor 50 min post injection revealed values of 3.0 ± 0.77 vs. 1.2 ± 0.77% ID/cc in the HCT116AF and HCT116, respectively (*p* < 0.01); (**C**) Dynamic uptake on tracer in HCT116 and HCT116AF tumors shows higher tracer uptake in AF over-expressing tumors at all time-points; (**D**) Treatment of mice (*n* = 2) with the non-radioactive reference compound appears to have a strong blocking effect, offering evidence that the tracer is specific to AF (** *p* < 0.01).

Although this work was primarily focused on tracer development, we evaluated the tracer in BALB/c nu/nu mice bearing HCT116AF (right) and HCT116 (left) xenografts. The xenograft model was complicated by the slow growth of the HCT116AF xenografts; static PET scans 50 min post injection revealed visibly higher activity in the transfected tumors compared to the controls (Figure 4A). Quantitative analysis (Figure 4B) of the PET data demonstrated 2.5-fold higher tracer uptake in the transfected tumors (3.0 ± 0.8%ID/cc (*n* = 6)) compared with the controls (1.2 ± 0.8 (*n* = 5)). Furthermore, dynamic scans of [^11^C]**3** (Figure 4C) were measured and showed the transfected tumors to have higher uptake than the untransfected tumors at all time-points (*n* = 2).

Owing to the complexity of the in vivo tumor growth and the primary focus on novel chemistry, blocking experiments (to assess specificity toward AF) were only performed in two mice (Figure 4D). Although a strong blocking effect was observed, this is only reported as a tendency.

## 3. Materials and Methods

### 3.1. General

#### 3.1.1. Chemistry

All reagents and solvents for synthesis, unless otherwise stated, were purchased from Sigma-Aldrich (Taufkirchen, Germany). Solvents and reagents were at least of analytical grade and were used without further purification. Dried solvents were obtained from Sigma-Aldrich and were stored under inert gas. Nuclear magnetic resonance (NMR) spectra were recorded on a Bruker Advance III 600 (151 MHz for ^13^C) spectrometer (Bruker, Ettlingen, Germany). NMR experiments were carried out in chloroform-d. Chemical shifts (δ) were recorded relative to residual chloroform (δ = 7.26 in ^1^H NMR and δ = 77.00 in ^13^C NMR). All chemical shifts are reported in ppm. High resolution mass spectroscopy (HRMS) was performed using electron spray ionization (ESI) and a MAXI4G spectrometer (Bruker Daltonics, Bremen, Germany). The accuracy, as designated in PPM, must be <2.00 from predicted mass. LCMS was performed using an Agilent 1200 series system and a 6120-quadrupole mass spectrometer (Agilent, Waldbronn, Germany), with a reverse-phase C18(2) analytical column (Phenomonex, Aschaffenburg, Germany; Luna C18(2) 100u, 250 × 4.6 mm, 5 μm). The mobile phase comprised CH_3_CN and water (+0.1% trifluoroacetic acid) and the flow rate was 1 mL/min. The standard gradient program started with 5% CH_3_CN for 2 min. The CH_3_CN component was then increased to 100% over 15 min, where it was maintained for a further 6 min. Mass spectra were obtained using ESI + APCI in positive and negative modes. 

*(2S,3S,4R,5S)-2-(4-Methoxyphenyl)-5-methylpyrrolidine-3,4-diol*, **3** [10]. The reference compound was synthesized in 9 steps from D-ribose according to the procedure outlined by Kotland et. al. TLC: R*f* = 0.55 (EtOAc:PE = 3:2); ^1^ HNMR (600 MHz, CDCl_3_) δ = 7.31 (d, *J* = 8.8 Hz, 2H, ArH), 6.87 (d, *J* = 8.8 Hz, 2H, ArH), 4.88 (d, *J* = 5.4 Hz, 1H, H-2), 4.50 (t, *J* = 5.4, 4.1 Hz, 1H, H-3), 4.30 (apparent s, 1H, H-1), 3.79 (s, 3H, OCH3), 3.12 (qd, *J* = 6.6, 4.1 Hz, 1H, H-4), 1.54 (s, 3H, CH3), 1.35 (s, 3H, CH_3_), 1.26 (d, *J* = 6.6 Hz, 3H, H-5); ^13^C NMR (151 MHz, CDCl_3_) δ = 158.46 (ArC), 127.86 (ArC), 113.80 (ArC), 113.18 (C_quat._), 110.87 (ArC), 88.66 (C-2), 83.37 (C-3), 66.13 (C-1), 56.66 (C-4), 55.25 (OCH3), 26.08 (CH3), 24.07 (CH3), 13.40 (C-5).

*4-((3aS,4S,6S,6aR)-2,2,6-Trimethyltetrahydro-4H-[1,3]dioxolo[4,5-c]pyrrol-4-yl)phenol*, **12** [10]. Compound **3** (84 mg, 0.32 mmol) was heated at 80 °C, in a mixture comprising 37% formaldehyde (1.4 mL) and formic acid (0.7 mL), until TLC revealed complete consumption of the starting material. The solution was diluted with saturated aqueous K_2_CO_3_ (10 mL) and extracted into DCM (4 × 15 mL). The combined organics were dried over MgSO_4_, and the solvent was removed under reduced pressure. Purification using silica gel column chromatography, with an eluent of 15% MeOH in DCM, yielded **12** as a white solid (75.0 mg, 85%). TLC: R*f* = 0.5 (EtOAc:PE = 1:1); ^1^HNMR (600 MHz, CDCl_3_) δ = 7.08 (d, *J* = 8.1 Hz, 2H, ArH), 6.66 (d, *J* = 8.1 Hz, 2H, ArH), 4.91 (d, *J* = 5.5 Hz, 1H, H-3), 4.67 (d, *J* = 5.1 Hz, 1H, H-2), 4.47 (apparent s, 1H, H-1), 3.48 (d, *J* = 1.8 Hz, 1H, H-4), 3.42 (t, *J* = 6.2 Hz, 1H, NH), 1.59 (s, 3H, CH3), 1.38 (d, *J* = 6.6 Hz, 3H, H-5), 1.34 (s, 3H, CH3); ^13^C NMR (151 MHz, CDCl_3_) δ = 156.29 (ArC_quat._), 130.09 (ArC_quat._), 128.75 (2 × ArC), 115.86 (2 × ArC), 111.68 (C_quat._), 86.34 (C-3), 81.71 (C-2), 66.27 (C-1), 56.72 (C-4), 25.95 (CH3), 24.01 (CH3), 12.70 (C-5); MS (ESI): [M + H]^+^(theor.) = 278.37, Measured = 278.0.

*(3aS,4S,6S,6aR)-5-Benzyl-4-(4-(benzyloxy)phenyl)-2,2,6-trimethyltetrahydro-4H-[1,3]dioxolo[4,5-c]pyrrole*, **7b**. The glycosylamine 5 (7.5 g, 28.7 mmol) was prepared according to the method of Kotland et. al. [10], dissolved in anhydrous THF (25 mL), and cooled to 0 °C under argon. p-benzyloxyphenylmagnesium bromide (1.0 M in THF, 100 mL) (Sigma Aldrich) was added through a dropping funnel over 10 min, and the reaction was warmed to room temperature and stirred for a further 16 h. After the reaction was quenched with saturated aqueous NH4Cl (200 mL), the material was extracted into Et2O (4 × 50 mL). The combined organic layers were washed with water (100 mL) and brine (100 mL), dried over MgSO4, and concentrated under vacuum. The crude material was flushed through a silica gel pad to yield the crude intermediate amino alcohol as a colorless oil (5.0 g). TLC: Rf = 0.25 (EtOAc:PE = 3:7)

Mesyl chloride (2.5 mL, 32.3 mmol) was added slowly to an ice-cooled solution of the intermediate material (4.5 g, 11.2 mmol) in pyridine (30 mL), under an argon atmosphere. After 30 min at 0 °C, the ice bath was removed, and the reaction was stirred for a further 6 h at room temperature. TLC analysis revealed the consumption of the starting material and the formation of a less polar product. The mixture was quenched with saturated aqueous NaHCO_3_ (50 mL), extracted into DCM (3 × 50 mL), and washed successively with NaHCO_3_ (50 mL), water (50 mL), and brine (50 mL). After drying over MgSO_4_ and concentrating under reduced pressure, the material was subjected to silica gel chromatography (10–40% EtOAc in PE). This yielded the **7b** as a colorless oil (2.1 g, 44%). TLC: R*_f_* = 0.5 (EtOAc:PE = 1:17); ^1^H NMR (600 MHz, CDCl_3_) δ = 7.47–7.26 (m, 10H, 10 × ArH), 7.09 (d, *J* = 8.6 Hz, 2H, ArH), 6.95 (d, *J* = 8.6 Hz, 2H, ArH), 5.06 (s, 2H, H-7), 4.79 (dd, *J* = 6.9, 5.9 Hz, 1H, H-3), 4.62 (dd, *J* = 6.9, 2.5 Hz, 1H, H-2), 3.98 (d, *J* = 2.4 Hz, 1H, H-1), 3.65 (d, *J* = 14.2 Hz, 1H, H-6_a_), 3.33 (*p*, *J* = 6.4 Hz, 1H, H-4), 3.28 (d, *J* = 14.2 Hz, 1H, H-6_b_), 1.61 (s, 3H, CH_3_), 1.35 (s, 3H, CH_3_), 1.10 (d, *J* = 6.5 Hz, 3H, H-5); ^13^C NMR (151 MHz, CDCl_3_) δ = 158.09 (ArCO_quat._), 139.52 (ArC_quat._), 136.91 (ArC_quat._), 131.83 (ArC_quat._), 129.44 (2 × ArC), 128.55 (2 × ArC), 128.11 (ArC), 128.10 (2 × ArC), 127.94 (ArC), 127.46 (2 × ArC), 126.56 (2 × ArC), 114.60 (2 × ArC), 112.41 (C_quat._), 86.18 (C-2), 80.79 (C-3), 69.95 (C-7), 69.11 (C-1), 57.06 (C-4), 50.25 (C-6), 26.24 (CH_3_), 25.17 (CH_3_), 10.60 (C-5); HRMS (ESI): [M + H]^+^ _(theor.)_ = 430.23767, measured = 430.23848

*(3aS,4S,6S,6aR)-5-Benzyl-4-(4-(benzyloxy)phenyl)-2,2,6-trimethyltetrahydro-4H-[1,3]dioxolo[4,5-c]pyrrole*, **8b**: To a 3-neck flask, charged with argon and cooled in an ice bath, was added Pd/C (10%, 150 mg). The catalyst was carefully solvated with EtOH (35 mL), after which **7b** (1.0 g, 2.3 mmol) in DCM (5 mL) was added. After repeatedly degassing the system (5×), the solution was placed under a H_2_ atmosphere and stirred overnight at room temperature. TLC analysis revealed the spot-to-spot conversion of the starting material to a more polar product. The solution was degassed, placed under an argon atmosphere, and filtered through Celite^®^ to remove the catalyst. The solvent was removed under reduced pressure, and the resulting colorless oil **8b** was used without further purification (0.4 g, 69%). TLC: R*_f_* = 0.35 (EtOAc:PE = 1:1); ^1^H NMR (600 MHz, CDCl_3_) δ = 7.08 (d, *J* = 8.1 Hz, 2H), 6.66 (d, *J* = 8.1 Hz, 2H), 4.91 (d, *J* = 5.5 Hz, 1H), 4.67 (d, *J* = 5.1 Hz, 1H), 4.47 (apparent s, 1H), 3.48 (d, *J* = 1.8 Hz, 1H), 3.42 (t, *J* = 6.2 Hz, 1H), 1.59 (s, 3H), 1.38 (d, *J* = 6.6 Hz, 3H), 1.34 (s, 3H); ^13^C NMR (151 MHz, CDCl_3_) δ = 156.29, 130.09, 128.75 (2 × ArC), 115.86 (2 × ArC), 111.68, 86.34, 81.71, 66.27, 56.72, 25.95, 24.01, 12.70; HRMS (ESI): [M + H]^+^ _(theor.)_ = 250.14377, measured = 250.14399.

*tert-Butyl (3aS,4S,6S,6aR)-4-(4-hydroxyphenyl)-2,2,6-trimethyltetrahydro-5H-[1,3]dioxolo[4,5-c]-pyrrole-5-carboxylate*, **10**: Boc_2_O (0.28 g, 1.3 mmol) in THF (1 mL) was added drop-wise to an ice-cooled solution of **8b** (214 mg, 0.86 mmol), Et_3_N (0.5 mL, 3.7 mmol), and NaHCO_3_ (0.3 g, 3.6 mmol) in THF (5 mL) and water (5 mL). The reaction was allowed to warm to room temperature over 1 h and was then stirred as such overnight. TLC analysis revealed the spot-to-spot conversion of the starting amine to the less polar carbamate. The solution was diluted with water (20 mL) and extracted into EtOAc (4 × 20 mL). The combined organic layers were washed with water (50 mL), dried over MgSO_4_, and concentrated under reduced pressure. Purification using silica gel column chromatography, which involved an increasing gradient of EtOAc in PE (20–50%), yielded the product **10** as a colorless syrup (0.20 g, 67%). TLC: R*_f_* = 0.53 (EtOAc:PE = 1:1); ^1^H NMR (600 MHz, CDCl_3_) δ = 6.92 (d, *J* = 8.5 Hz, 2H), 6.72 (d, *J* = 8.5 Hz, 2H), 4.84 (s, 1H), 4.81–4.73 (m, 1H), 4.50 (d, *J* = 6.1 Hz, 1H), 4.27–4.18 (m, 1H), 4.00–3.91 (m, 1H), 1.54–1.10 (m, 18H); HRMS (ESI): [M + Na]^+^ _(theor.)_ = 372.17814, measured = 372.17823

Acid hydrolysis of acetonide: A solution of **12** (5 mg, 18.0 μmol), acetonitrile (1 mL), and HCl (6.0 M, 150 μL) was heated at 140 °C for 5 min in a sealed flask. Upon cooling, the solution was analyzed using LCMS. (ESI-APCI): **13** (9.5 min), [M + H]^+^_(theor.)_ = 278.37, Measured = 278.0; **13** (5.3 min), [M + H]^+^_(theor.)_ = 238.31, measured = 238.1.

#### 3.1.2. Radiochemistry and Tracer Validation

^11^C was produced through the ^14^N(p,a)^11^C nuclear reaction as [^11^C]CO_2_, using a 16.5 MeV PETtrace cyclotron (GE Healthcare, Uppsala, Sweden), from N_2_ that contained 0.5% (*v*/*v*) O_2_. The beam current was 70 μA. [^11^C]CH_3_I was produced using an automated Tracerlab FX-MeI synthesis module (GE Healthcare). The automated synthesis was performed by an FX-M synthesis module (GE Healthcare). Radio-HPLC was performed using a 1260 Infinity HPLC system (Agilent) with a thallium activated sodium iodide scintillation detector. The column and gradient were the same as described for LCMS. Centrifugation for serum stability was performed using an Eppendorf mini-spin (Hamburg, Germany).

A suspension of NaH (5 mg) and the precursor **10** (10 mg) in anhydrous CH_3_CN was prepared and ultra-sonicated for 5 min at room temperature. This solution was then transferred directly to the reactor of the FX-M synthesis module, and cooled to −20 °C. The [^11^C]CH_3_I was transferred through a PTFE delivery line and trapped in the reactor at −20 °C. Upon completion of delivery, the reactor was heated to 110 °C for 5 min and then cooled to 40 °C. Next, 6.0 M HCl (200 μL) was added, and the reactor was heated to 140 °C for 5 min to allow for the removal of the carbamate and hydrolysis of the acetonide protection. The reactor was then cooled to 40 °C, after which a solution comprising 2.0 M NH_4_OAc (1.1 mL) and 6.0 M NaOH (0.2 mL) was added. The product was purified with semi-preparative HPLC (5.0 mL/min, Phenomenex, Luna C18(2), 100u, 250 × 10.0 mm, 5 μm), using 10% EtOH in PBS (pH 6.0) as eluent. The identity of the tracer was confirmed using radio-HPLC, by spiking with the authentic nonradioactive standard **3** that was synthesized according to the method outlined by Kotland et al. [10]. A calibration curve made from measuring dilutions of **3** at 254 nm was used to calculate the molar activity. A mixture of tracer (200 μL) and mouse serum (200 μL, Balb/C) was incubated with gentle agitation at 37 °C for 60 min. Cold acetonitrile (800 μL) was added, after which the mixture was vortexed and placed on ice for 5 min. Centrifugation at 10,000 rpm for 5 min yielded a supernatant that was analyzed by radio-HPLC.

#### 3.1.3. Cell Culture and Western Blot

Cells were grown in CELLSTAR^®^ standard cell culture flasks (Greiner Bio-One GmbH, Frickenhausen, Germany) at 37 °C in a humidified atmosphere of 5% CO_2_. HCT116 cells were cultured in McCoy’s 5A medium (ThermoFisher Scientific, Karlsruhe, Germany). Growth medium was supplemented with penicillin (100 U/mL), streptomycin (100 mg/L), and 10% fetal calf serum (FCS), and cells were regularly tested for mycoplasma using Venor GeM Mycoplasma Detection Kit.

pCMV6 mammalian vector containing cDNA of human α-fucosidase (FUCA1) was purchased from OriGene (Herford, Germany). The plasmid was amplified in NEB Turbo cells, harvested using a Midi-Prep Plasmid kit (QIAGEN GmbH, Hilden, Germany), and verified by Sanger sequencing. Cells were stably transfected using Lipofectamine^®^ 3000 (ThermoFisher) and treated in accordance with the manufacturer’s guidelines, using G418 selection reagent (ThermoFisher). Clones were isolated by selective trypsination and screened individually for FUCA1 expression by Western blot.

Cells or tissue were suspended in a mixture comprising lysis buffer (200 μL) and cOmplete™ protease inhibition buffer (ThermoFisher; 1 tablet in 7 mL PBS). The mixture was mechanically triturated at 0 °C for 5 min, after which it was centrifuged (10,000× *g*) for 5 min at 4 °C. A mixture of the lysate (20 μL) and 2× sample loading buffer (20 μL) was heated to 95 °C for 5 min and then loaded onto an Expedeon RunBlue—20% precast gradient gel (Abcam, Cambridge, UK). The separation was effected by running at 60 V for 10 min, followed by 150 V for 40 min. The proteins were transferred to a PVDF membrane using a Trans-Blot^®^ Transfer system (Biorad, Feldkirschen, Germany). The membrane was incubated for 1 h in Odyssey PBS blocking buffer (Li-Cor, Bad Homborg vor der Hoehe, Germany). Protein bands were stained with primary mouse anti-DDK2 (1:2000, Origene) and rabbit anti-β-actin (1:3333, Cell Signaling Technology, Leiden, The Netherlands) antibodies. After incubation with IRDye 680RD goat (polyclonal) anti-mouse (1:15,000, Li-Cor) and IRDye 800CW Goat (polyclonal) anti-rabbit (1:15,000, Li-Cor) secondary antibodies, the readout was performed using the Odyssey Sa imaging system (Li-Cor).

#### 3.1.4. In Vitro Evaluation

HCT116AF (overexpressing FUCA1) and HCT116 were grown to 70–80% confluence. The medium was removed, and cells washed with PBS. They were incubated with 10 MBq tracer in PBS containing 0.1% FCS (10 mL)) for 20 min at 37 °C. The cells were washed with PBS, detached with trypsin, and counted. One million cells were then aliquoted into each gamma-counter tube. Uptake was quantified using a standard curve with known activity with the data of the gamma counter Wizard2 2480 (Perkin Elmer, Waltham, MA, USA) after decay corrected activity detection. All data were normalized to the number of cells in each tube.

#### 3.1.5. Ex Vivo Evaluation

Tumor cryosections (10 μm) on microscope slides were fixed for 10 min at room temperature in 3.7% paraformaldehyde, after which they were washed with PBS (2 × 5 mL). The samples were then incubated for 5 min in PBS containing Triton X-100 (10 mL, 1:1000) to permeablize the cells. Blocking was performed by incubating the samples for 20 min in 1% BSA in PBS, which was followed by washing with PBS (2 × 5 mL). Staining involved the same primary antibodies used for Western blot. Thus, the slides were incubated first with the primary anti-DDK antibody (1:1000) for 1 h at room temperature, followed by a 1 h incubation with the secondary antibody anti-mouse IgG Alexa Fluor^®^ 555 Conjugate (1:1000, Cell Signaling Technology). Nuclear counterstaining was performed by incubating the samples in 300 nM DAPI (ThermoFischer Scientific) for 5 min and washing thoroughly with PBS (3 × 5 mL). The slides were carefully cleaned, mounted with Mowiol^®^ 4-88 mounting medium (Sigma Aldrich), and examined under a Axiovert 200 inverted fluorescence microscope (Carl Zeiss, Oberkochen, Germany).

#### 3.1.6. PET Studies and Blocking

Animal studies were conducted according to approved animal use and care protocols in accordance with the German Animal Protection Law (Regierungspräsidium Tübingen). For in vivo experiments, tumors were induced in the flank of BALB/c nu/nu mice by subcutaneous injection of 1 × 10^6^ HCT116AF (left) and HCT116 (right) cells. For in vivo experiments, tumors achieved a volume of approximately 250–300 mm³. A calibrated vaporizer (Vetland, Louisville, KY, USA) was used to anesthetize the mice at 1.5% (*v*/*v*) isoflurane (Forene, Abbott Labs, Baar, Switzerland) in oxygen. Anesthesia was monitored by measuring the respiratory frequency, and the body temperature was kept at 37 °C using a heating pad. The radiolabeled AF inhibitor (12.8 ± 1.7 MBq) was injected into the tail vein. PET data were acquired in list-mode, histogrammed, and reconstructed by using an iterative ordered subset expectation maximization (OSEM) algorithm. No attenuation correction was applied. MR imaging was performed on a 7 T small animal MR tomograph (Clinscan, Bruker Biospin MRI), obtaining anatomical information for organ delineation. A T2WI 3D space sequence (TE/TR 202/2500 ms, image matrix of 137 × 320, slice thickness 0.27 mm) was used for whole-body imaging. PET images were normalized to each other, subsequently fused to the respective MR images, and analyzed by using Inveon Research Workplace software (Siemens Preclinical Solutions). Regions of interest (ROIs) were drawn around the whole tumor and reference (muscle) tissue based on the anatomical information from the MR images. Absolute quantification of the PET data was expressed as percentage of the injected dose per cubic centimeter (%ID/cc).

We performed a preliminary blocking experiment using just 2 mice that had a similar size and stage of tumor. Blocking was performed by injecting 5 μL of a 3.5 mg/mL solution of non-radioactive compound **3** in PBS 10 min prior to administering the tracer.

#### 3.1.7. Statistics

All statistics were performed using Graphpad Prism 7. All data sets were checked for normality using the D’Agostino-Pearson test. The comparison of multiple (*n* > 2) groups relied on the Kruskal–Wallis test when data were not normally distributed. When data sets passed the normality test, analysis of variance (ANOVA) was used. Comparing the differences between 2 groups relied on either a two-tailed t test or Mann–Whitney test. Differences were deemed significant when *p* < 0.05 and highly significant when *p* < 0.01.

## 4. Conclusions and Outlook

In our efforts to develop a probe for AF, we successfully synthesized a precursor that is suitable for late-stage ^11^C methylation and rapid deprotection. We further produced an AF overexpressing cell model, which could be translated for preclinical imaging. Our results evidence a probe that has the ability to identify and visualize the expression of AF both in vitro and in vivo. Future work will focus on applying this imaging probe to various disease models, including arthritis and cancer.

## Data Availability

Data is contained within the article and supplementary material.

## Supplementary Materials

The following are available online at https://www.mdpi.com/article/10.3390/ph14080745/s1, Figure S1: 1^1^ H NMR of compound **7b**, 2^13^ C NMR of compound **7b**, Figure S2: 1^1^ H NMR of compound **8b**, 2^13^C NMR of compound **8b**, Figure S3: 1^1^ H NMR of compound **10**, 2^13^ C NMR of compound **10**, Figure S4: HRMS of compound **10**, Figure S5: Semipreparative HPLC chromatogram of [^11^C]3 from GE FX Module.
