# Supplementary material for: Synthesis and Biological Evaluation of a Radiolabeled PET (Positron Emission Tomography) Probe for Visualization of In Vivo α-Fucosidase Expression
Source: Pharmaceuticals (Basel). 2021 Jul 29;14(8):745. doi: 10.3390/ph14080745 (PMC8402005; doi:10.3390/ph14080745)

Supplementary Materials

Figure S1.A. ^{1}H NMR of compound **7b**

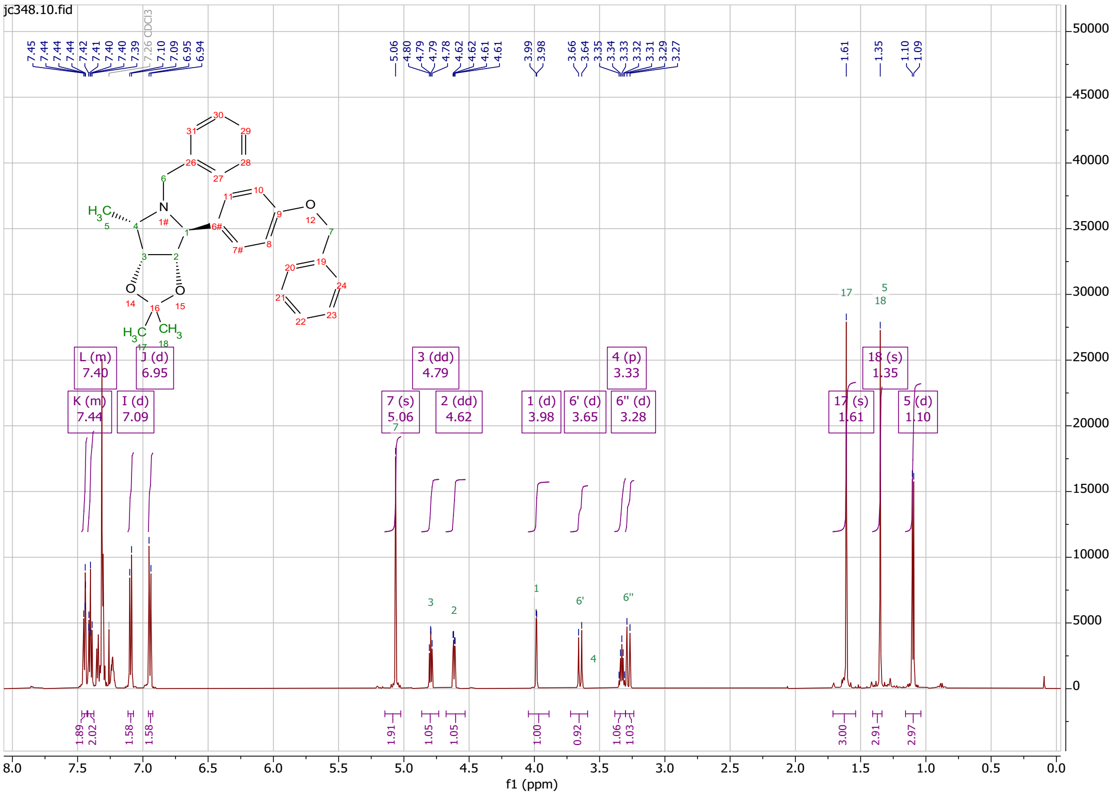

Figure S1.B. ^{13}C NMR of compound **7b**

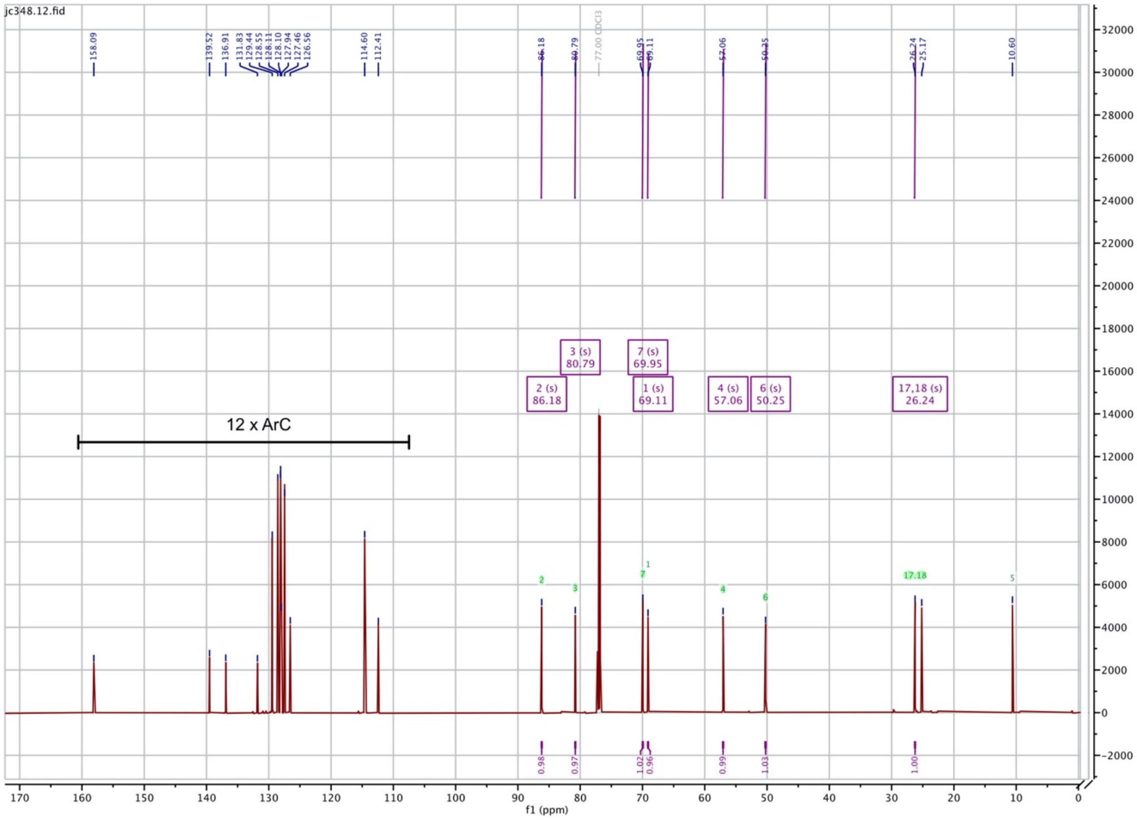

Figure S2.A. ^{1}H NMR of compound **8b**

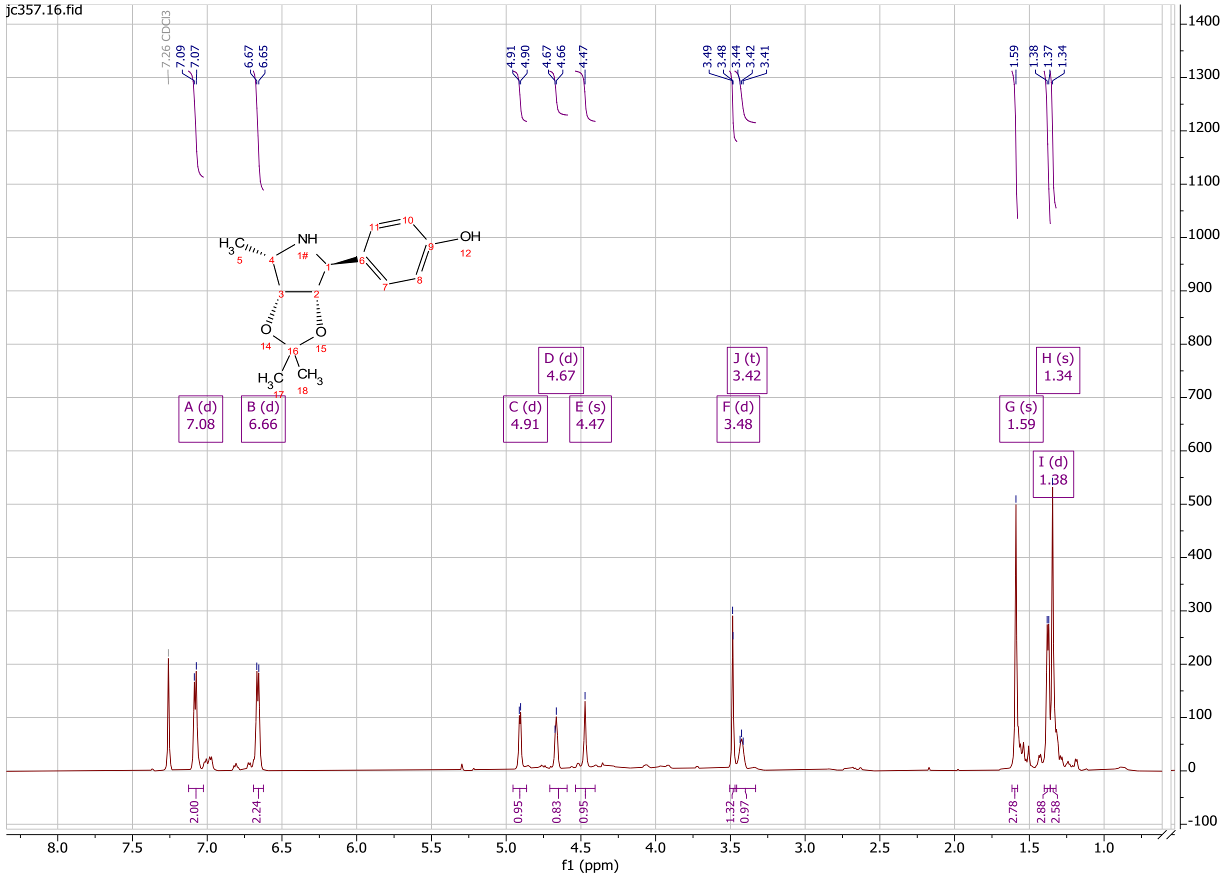

Figure S2.B. ^{13}C NMR of compound **8b**

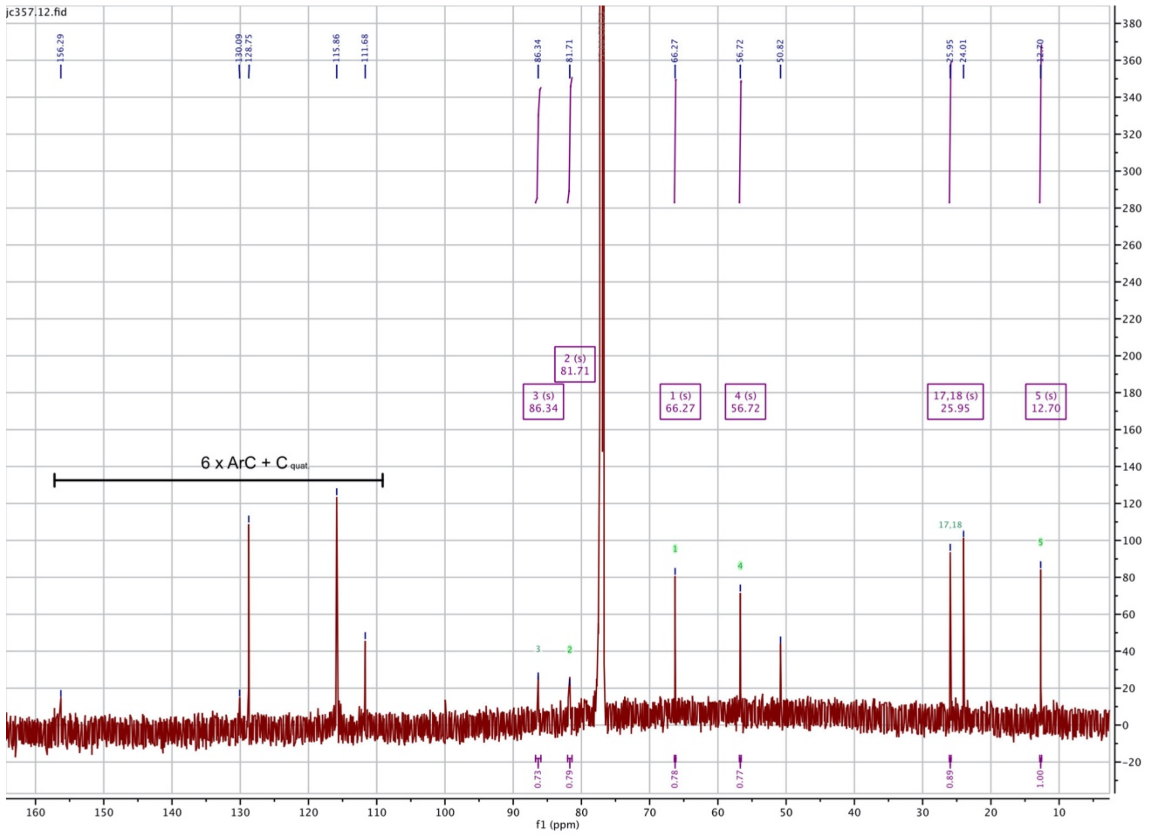

Figure S3.A. ^{1}H NMR of compound **10**

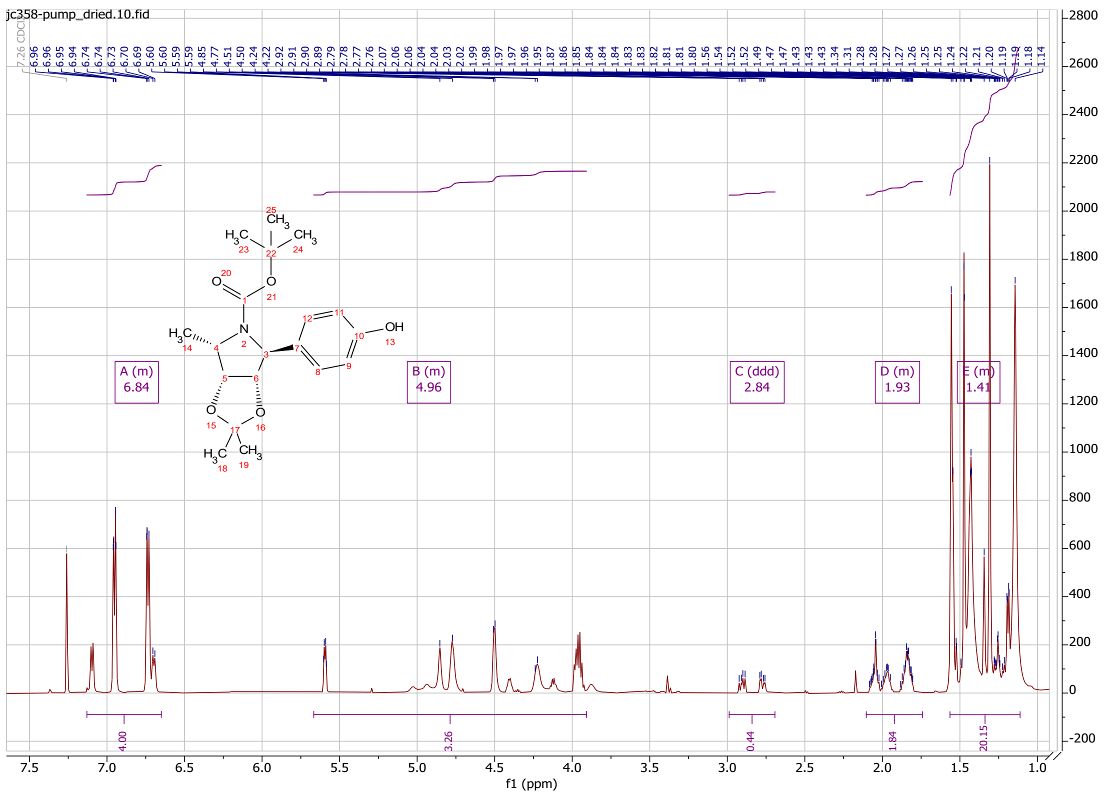

Figure S3.B. ^{13}C NMR of compound **10**

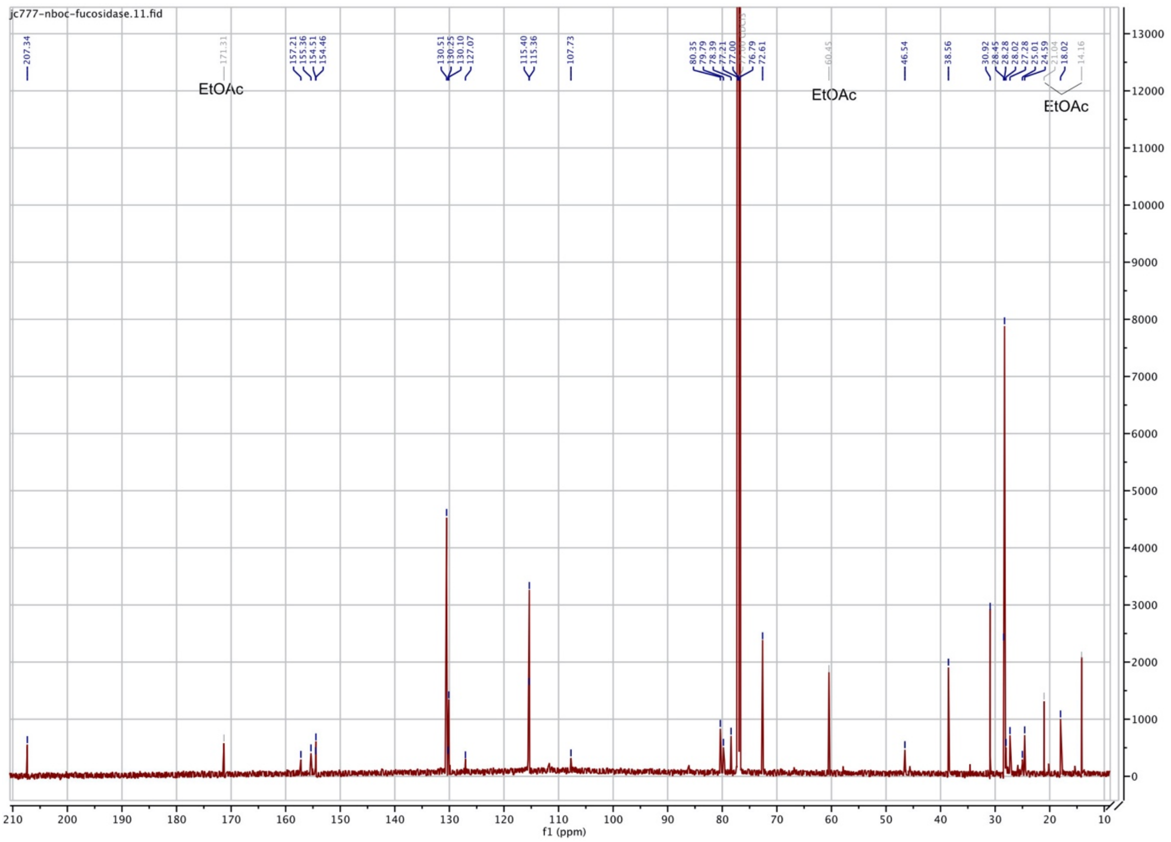

Figure S4. HRMS of compound **10**

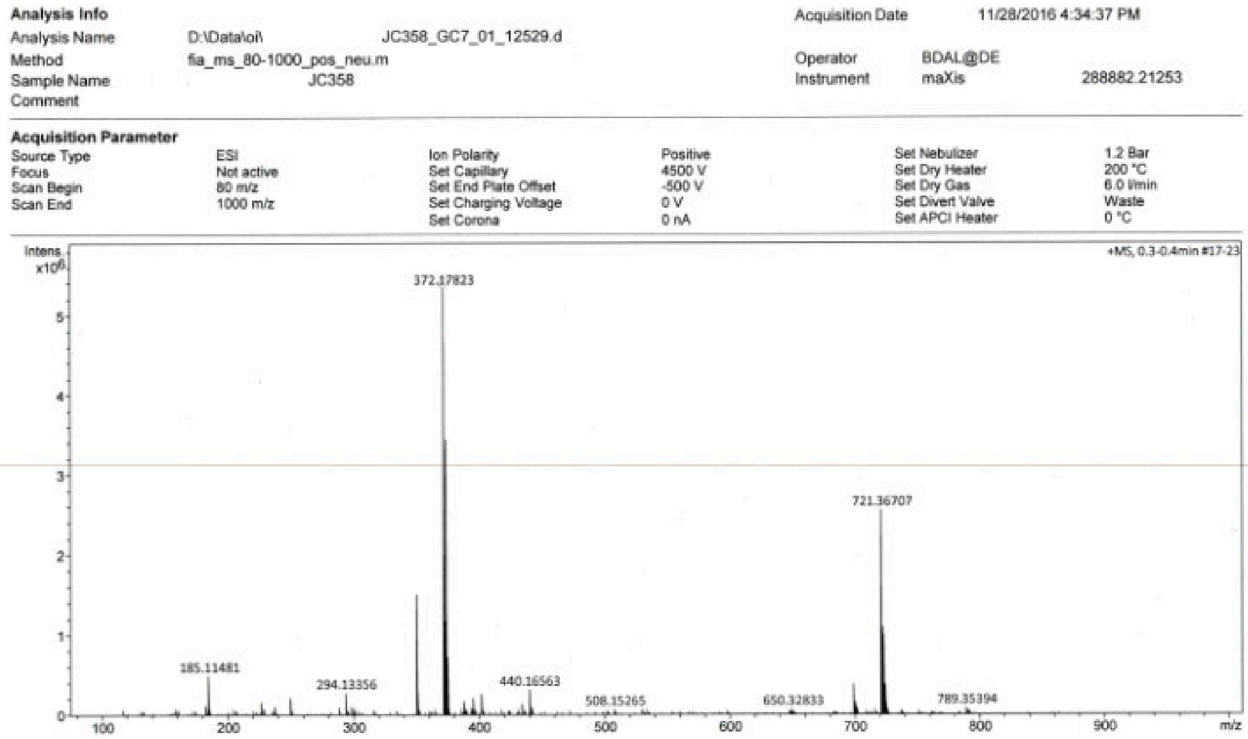

Figure S5. Semipreparative HPLC chromatogram of [^{11}C]**3** from GE FX Module

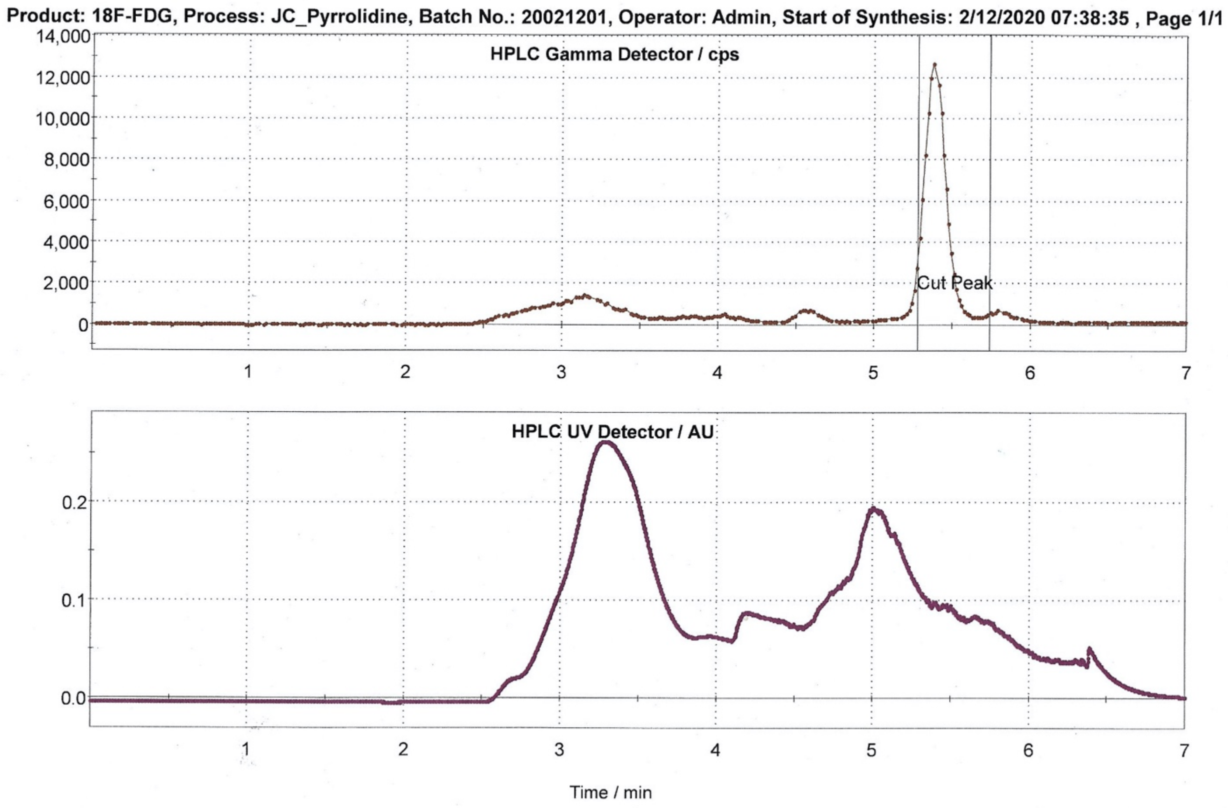

Supplement: Supplementary file 1 [file pharmaceuticals-14-00745-s001.zip › pharmaceuticals-1298445-supplementary.pdf]
